# Association of serum lipids with β-cell function in obese children and adolescents

**DOI:** 10.1530/EC-19-0333

**Published:** 2019-09-13

**Authors:** Giorgio Bedogni, Andrea Mari, Alessandra De Col, Sofia Tamini, Amalia Gastaldelli, Alessandro Sartorio

**Affiliations:** 1 Liver Research Center, Basovizza, Trieste, Italy; 2 Institute of Neuroscience, National Research Council, Padova, Italy; 3 Istituto Auxologico Italiano, IRCCS, Experimental Laboratory for Auxo-Endocrinological Research, Piancavallo (VB), Italy; 4 Institute of Clinical Physiology, National Research Council, Pisa, Italy; 5 Istituto Auxologico Italiano, IRCCS, Division of Metabolic Diseases and Auxology, Piancavallo (VB), Italy

**Keywords:** obesity, children, adolescents, insulin secretion, triglycerides, cholesterol

## Abstract

Few data are available on the association between serum lipids and insulin secretion (ISEC) in children. We evaluated the association of triglycerides (TG), HDL cholesterol (HDL-C) and LDL cholesterol (LDL-C) with ISEC in 1150 non-diabetic obese children and adolescents using multivariable robust median regression. The following models were employed: (1) IGI or incAUCR as the ISEC response variable; (2) QUICKI, OGIS, the Stumvoll index or the Matsuda insulin sensitivity index as the insulin sensitivity (ISEN) predictor; (3) TG, HDL-C and LDL-C as the predictors of interest; (4) 120-min glucose, age, sex and body mass index as confounders. LDL-C and TG were not associated with ISEC in any model. In three out of four IGI models, an increase of 1 interquartile range (IQR) of HDL-C was associated with a decrease of median incAUCR ranging from −9 (robust 95% CI −17 to −2) to −8 (−14 to −1) pmol/mmol. In two out of four incAUCR models, an increase of 1 IQR of HDL-C was associated with a decrease of median IGI ranging from −8 (−15 to −1) to −7 (−11 to −2) pmol/mmol. TG and LDL-C are not associated and HDL-C is inversely associated with ISEC in obese children and adolescents.

## Introduction

The prevalence of childhood type 2 diabetes mellitus (T2DM) is rapidly increasing worldwide (1). T2DM is characterized by visceral obesity, insulin resistance and defective β-cell function. In children and adolescents, glucose metabolism deteriorates more rapidly than in adults but with similar mechanisms (1).

The RISC (Relationship between Insulin Sensitivity and Cardiovascular risk) study has recently evaluated the association between serum lipids and β-cell function under the hypothesis that lipids contribute to glucose intolerance (2). Accumulation of toxic lipid products in muscle, liver, adipocytes and β-cells is in fact likely to contribute to insulin resistance and β-cell dysfunction (3). In detail, RISC has shown that insulin secretion (ISEC) is independently associated with triglycerides (TG) and high-density lipoprotein cholesterol (HDL-C) but not with low-density lipoprotein cholesterol (LDL-C) (2). As noted by the RISC investigators (2), most of the previous studies focused on the association of free-fatty acids (FFA) with β-cell function and did not evaluate the association of this latter with TG, HDL-C and LDL-C (2).

A situation similar to that described by the RISC investigators for adults (2) is apparent in children and adolescents, where most of the available studies focused on the association between FFA and β-cell function, and no systematic investigation is presently available on the relationship between β-cell function and TG, HDL-C and LDL-C (4, 5).

The aim of the present study was, therefore, to evaluate the multivariable association of β-cell function with TG, HDL-C and LDL-C in a large sample of non-diabetic obese children and adolescents taking into account known confounders.

## Materials and methods

### Study design

A retrospective cross-sectional study was performed on 1150 consecutive children and adolescents followed at our Pediatric Obesity Clinic between January 2009 and March 2014. They were admitted to the clinic to undergo a short-term structured multidisciplinary weight-loss program. The inclusion criteria for the present study were (1) age ≥5 and age ≤18 years; (2) body mass index (BMI) ≥95^th^ percentile for age and sex using Italian growth data (6); (3) availability of oral glucose tolerance test (OGTT); (4) absence of type 1 or type 2 diabetes mellitus. The exclusion criteria were (1) genetic or syndromic obesity; (2) treatment with drugs known to interfere with glucose metabolism. The study was approved by the Ethical Committee of the Istituto Auxologico Italiano (Code: 01C725; acronym: TGHDLOBES) and was conducted in accordance with the 1975 Declaration of Helsinki, as revised in 2008. The parents or the legal guardians of the subjects or the subjects themselves when aged 18 years gave the written informed consent to participate to the study.

### Clinical and anthropometric assessment

Pubertal status was classiﬁed in five stages according to Tanner (7). Weight and height were measured following standard procedures (8). BMI was calculated as weight (kg)/squared height (m^2^). Standard deviation scores (SDS) of weight, stature, and BMI were calculated using Italian growth data (6).

### Oral glucose tolerance test

Glucose tolerance was assessed by OGTT with 1.75 g of glucose per kg of weight (up to 75 g) (9). Glucose and insulin were measured at 0, 30, 60, 90, and 120 min during OGTT. Glucose was measured using standard laboratory methods, and insulin was measured using a chemiluminescent immunoassay (Immulite 2000, Diagnostic Products Corporation, Los Angeles, CA, USA). T2DM was deﬁned as 120-min-OGTT glucose ≥11.1 mmol/L and impaired glucose tolerance (IGT) as 120-min-OGTT glucose ≥7.8 mmol/L and <11.1 mmol/L (9).

### Calculation of the indices of insulin secretion and sensitivity

Two indices of ISEC were calculated: the insulinogenic index (IGI), that is, the ratio of the increments from 0 to 30 min of insulin and glucose (10, 11), and the ratio between the incremental areas under the curve of insulin and glucose (incAUCR) (10, 11). Four indices of ISEN were calculated: (1) the quantitative insulin sensitivity check index (QUICKI) (12); (2) the oral glucose insulin sensitivity index at 2 h (OGIS) (13); (3) the Stumvoll index (SI) (14); (4) the Matsuda insulin sensitivity index (ISI) (15). IncAUCR, OGIS, SI, and ISI were calculated from glucose and insulin expressed as international units. Insulin was converted from standard (μU/mL) to international (pmol/l) units using a conversion factor of 6.0 (16).

### Statistical analysis

Most continuous variables were non-Gaussian distributed and all are reported as 50^th^ percentile (median) and 25^th^ and 75^th^ percentiles. Discrete variables are reported as counts and proportions. Pre-specified multivariable robust median regression models were used to evaluate the association between ISEC and serum lipids (17, 18). Such models employed: (1) IGI or incAUCR as the ISEC continuous response variable; (2) QUICKI, OGIS, SI or lnISI (natural logarithm of ISI) as the ISEN continuous predictor; (3) TG (continuous), HDL-C (continuous) and LDL-C (continuous) as the predictors of interest; (4) 120-min-glucose (continuous), age (continuous), sex (discrete: 0 = female; 1 = male) and BMI SDS (continuous) as predictors to be accounted for as known confounders. We used multivariable fractional polynomials (MFP) to test whether the multivariable relationships of the continuous predictors with ISEC were linear and to transform the predictors to achieve linearity when needed (19). We log-transformed ISI using natural logarithms on the basis of its univariable associations with both IGI and incAUCR. This transformation was not improved to a practically relevant degree by MFP and all the other continuous predictors showed a linear relationship with the outcome at multivariable analysis. Each continuous predictor was divided by its interquartile range (IQR, difference between 75^th^ and 25^th^ percentile) to obtain standardized regression coefficients representing a similar effect size (20). Statistical analysis was performed using Stata 15.1 (Stata Corporation).

## Results

Table 1 gives the measurements of the 1150 obese children and adolescents.

**Table 1:** Measurements of the children and adolescents.

	*n* = 1150
Glucose tolerance	
Normal glucose tolerance	1008 (87.7%)
Impaired glucose tolerance	142 (12.3%)
Sex	
Girls	685 (59.6%)
Boys	465 (40.4%)
Age (years)	15 (13–17)
Pubertal stage (Tanner)	
1	107 (9.3%)
2	91 (7.9%)
3	140 (12.2%)
4	254 (22.1%)
5	558 (48.5%)
Weight (kg)	96.4 (84.8–112.1)
Weight (SDS)	3.08 (2.57–3.63)
Height (m)	1.63 (1.56–1.69)
Height (SDS)	0.11 (−0.52, 0.89)
BMI (kg/m^2^)	36.3 (32.8–40.2)
BMI (SDS)	3.03 (2.64–3.41)
Glucose 0 min (mmol/L)	4.4 (4.1–4.6)
Glucose 30 min (mmol/L)	6.5 (5.8–7.2)
Glucose 60 min (mmol/L)	7.0 (6.2–7.8)
Glucose 90 min (mmol/L)	6.7 (6.0–7.5)
Glucose 120 min (mmol/L)	6.4 (5.7–7.1)
Insulin 0 min (pmol/L)	75 (55–101)
Insulin 30 min (pmol/L)	356 (245–517)
Insulin 60 min (pmol/L)	410 (297–563)
Insulin 90 min (pmol/L)	422 (298–577)
Insulin 120 min (pmol/L)	427 (307–595)
IGI (pmol/mmol)	137 (96–205)
incAUCR (pmol/mmol)	150 (109–200)
QUICKI (dimensionless)	1.87 (1.81–1.95)
OGIS (ml/min/m^2^)	443 (411–476)
SI (μmol/kg/min/pmol/L)	0.08 (0.06–0.09)
ISI (μmol/kg/pmol)	11.9 (8.9–15.7)
LnISI	2.47 (2.19–2.75)
Triglycerides (mmol/L)	1.0 (0.8–1.3)
Cholesterol (mmol/L)	4.2 (3.7–4.7)
HDL cholesterol (mmol/L)	1.1 (1.0–1.3)
LDL cholesterol (mmol/L)	2.6 (2.2–3.2)

BMI, body mass index; IGI, insulinogenic index; incAUCR, ratio between the incremental areas under the curve of insulin and glucose; ISI, Matsuda insulin sensitivity index; Ln, natural logarithm; OGIS, insulin sensitivity index at 2 h; QUICKI, quantitative insulin sensitivity check index; SDS, standard deviation scores; SI, Stumvoll index (SI).

**Figure 1:**
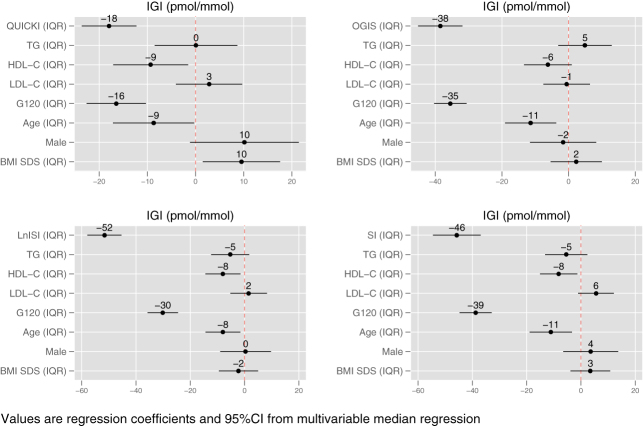
Standardized regression coefficients and 95% confidence intervals of the four multivariable median regression models using IGI as measure of insulin secretion (ISEC). Standardization was obtained by dividing each continuous predictor by its interquartile range. BMI SDS, standard deviation score of body mass index; G120, glucose at 120 min; HDL-C, HDL cholesterol; IGI, insulinogenic index; IQR, interquartile range; LDL-C, LDL cholesterol; LnISI, natural logarithm of Matsuda insulin sensitivity index; OGIS, oral glucose insulin sensitivity index at 2 h; QUICKI, quantitative insulin sensitivity check index; SI, Stumvoll index; TG, triglycerides.

**Figure 2:**
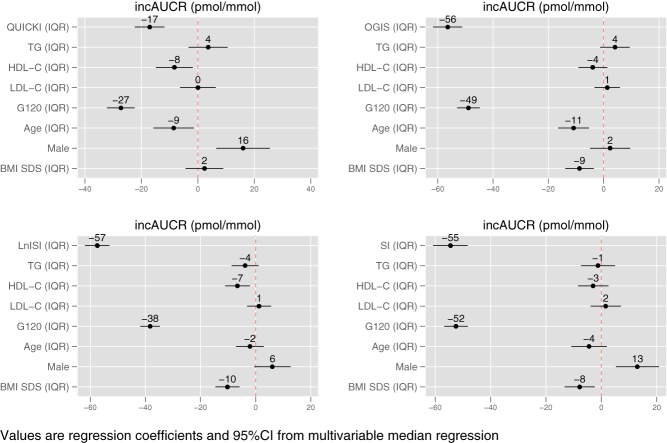
Standardized regression coefficients and 95% confidence intervals of the four multivariable median regression models using incAUCR as measure of insulin secretion (ISEC). Standardization was obtained by dividing each continuous predictor by its interquartile range. BMI SDS, standard deviation score of body mass index; G120, glucose at 120 min; HDL-C, HDL cholesterol; incAUCR, incremental area under the curves of insulin and glucose; IQR, interquartile range; LDL-C, LDL cholesterol; LnISI, natural logarithm of Matsuda insulin sensitivity index; OGIS, oral glucose insulin sensitivity index at 2 h; QUICKI, quantitative insulin sensitivity check index; SI, Stumvoll index; TG, triglycerides.

Figure 1 plots the standardized regression coefficients and the 95% confidence intervals of the four multivariable median regression models using IGI as measure of ISEC and Fig. 2 does the same for the four models using incAUCR as index of ISEC.

Expectedly (17), the greatest contribution to ISEC in all models came from the ISEN indexes and glucose at 120 min.

TG were not associated with ISEC in any model and the same was true for LDL-C, while HDL-C was associated with ISEC in three out of four IGI models and in two out of four incAUCR models.

In the three IGI models, an increase of 1 IQR of HDL-C was associated with a decrease of median IGI ranging from −9 (robust 95% CI −17 to −2, *P* < 0.05) to −8 (−14 to −1, *P* < 0.05) pmol/mmol.

In the two incAUCR models, an increase of 1 IQR of HDL-C was associated with a decrease of median incAUCR ranging from −8 (−15 to −1, *P* < 0.05) to −7 (−11 to −2, *P* < 0.01) pmol/mmol.

## Discussion

This is the first study to systematically investigate the relationship between β-cell function and serum lipids in obese children and adolescents. Eight ISEC models were used as surrogate measures of β-cell function (17) and to evaluate the independent effect of TG, LDL-C and HDL-C on ISEC taking into account ISEN, glucose at 120 min, age, sex and BMI. All continuous predictors were modeled as such and transformed using MFP to avoid problems with categorizations (21). In the present study, TG and LDL-C were not associated with ISEC in any model, while HDL-C was associated with ISEC in five out of eight models.

In contrast to the RISC study (2), where an association was detected between ISEC and TG, we did not find any association between ISEC and TG in any model. More important than the lack of statistical significance, also because we did not prespecify a null hypothesis (22), is the fact that the standardized coefficient of TG changed sign across models and that its effect size was always low both in absolute terms and in comparison with the standardized coefficients of the other predictors. (The stability of the sign of the coefficients across models can be taken as a very minimal criterion of coherence of the findings.).

In agreement with the RISC study (2), we did not find any association between ISEC and LDL-C. As discussed earlier for TG, more important than the lack of statistical significance, also because we did not prespecify any null hypothesis (22), is the fact the standardized coefficient of LDL changed sign across models and that its effect size was always low both in absolute terms and in comparison with the coefficients of the other predictors. As pointed out by the RISC investigators (2), these findings are at odds with those of experimental animal studies showing that an experimentally induced increase of LDL-C can reduce glucose-induced ISEC in a biologically relevant way. A great advantage of experimental studies is that they can evaluate the casual effect of a treatment (e.g. LDL-C increase) on a given outcome (e.g. ISEC). Extrapolating experimental data from animals to humans is, however, fraught with difficulties, especially when the data available for human are based on observational studies and some experiments may be unfeasible or unethical to perform.

Still in agreement with the RISC study (2), an inverse association between ISEC and HDL-C was found (five out of eight regression models).

In the IGI models, an increase of 1 IQR of HDL-C was associated with a decrease of median IGI ranging from −9 to −8 pmol/mmol. This effect size corresponds to 6–7% of median IGI. Although this contribution is possibly too low to be biologically relevant, it is of interest that HDL-C was often among the strongest predictors of IGI. The reason why we are not sure of the biological relevance of this finding is the fact that the point estimate of the effect size corresponding to a change of 1 IQR of HDL may be lower than the measurement error of OGTT (23), from which IGI is obtained. As the relative ranking of predictors is concerned, taking for instance the IGI model using QUICKI as index of ISEN, the effect size of HDL-C was 50% of that of QUICKI (best predictor) and 56% of that of glucose at 120 min (second best predictor). It is also of interest that the absolute effect size of HDL-C on IGI remained similar in all models differently from what happened from most predictors.

It is likewise noteworthy that the sign of the association between incAUCR and HDL-C was negative in all models, although statistical significance was reached in just two models. In these two models, an increase of 1 IQR of HDL-C was associated with a decrease of median incAUCR ranging from −8 to −7 pmol/mmol. This effect corresponds to 4–5% of median incAUCR. Although this contribution is possibly too low to be biologically relevant, it is of interest that HDL-C often ranked among the first predictors of IGI. As stated earlier, the reason why we are not sure of the biological relevance of this finding is the fact that the point estimate of the effect size corresponding to a change of 1 IQR of HDL may be lower than the measurement error of OGTT, from which incAUCR is obtained (23). As the relative ranking of predictors is concerned, taking for instance the incAUCR model using QUICKI as index of ISEN, HDL-C had an effect size corresponding to 30% of that of glucose at 120 min (first predictor) and 47% of that of QUICKI (second best predictor). As the association of HDL with ISEC is concerned, it is noteworthy that HDL is a known index of insulin sensitivity and is associated with impaired suppression of endogenous glucose production (24, 25).

Even if this is the first study to systematically investigate the relationship between β-cell function and serum lipids in obese children and adolescents, it has nonetheless some limitations. The first limitation is that ISEC was evaluated using surrogate indices. Although these indices are clearly the most feasible option for epidemiological studies (17) and although the eight different ISEC models gave generally consistent results, only the use of a reference method to measure β-cell function can shed definitive light on the ISEC of obese children and adolescents (26). For instance, the RISC researchers employed a model-based assessment of ISEC based on C-peptide, rather than insulin, which accounts for insulin clearance (2, 27). Unfortunately, in the present study, C-peptide was not measured. The second limitation is that, even if our data are mostly concordant with those obtained by the population-based RISC study, which used a more sophisticated index of ISEC (2), they were obtained in a sample of severely obese children and adolescents followed at a tertiary care center and may therefore not extend to the general population. The third limitation is that the precision of the estimates, as detected by the 95% CI, was low in all cases. For instance, the greatest effect of the increase of 1 IQR of HDL-C on IGI was −9 (robust 95% CI −17 to −2). This 95% CI interval is compatible with anything from a fair effect (−17%) to virtually no effect (−2%). Because of the large number of subjects enrolled in the present study, this imprecision is likely to stem more from systematic rather than from random variation (23).

In conclusion, in a large sample of severely obese children and adolescents followed at a Pediatric Obesity Center, TG and LDL-C were not associated with ISEC, while HDL-C was inversely associated with ISEC taking into account ISEN, glucose at 120-min, age, sex and BMI. We believe that the next step to be taken to evaluate the biological relevance of the inverse association between ISEC and HDL-C that we detected in our severely obese children, should be testing for its existence on cross-sections including normal-weight, overweight and not severely obese children in addition to severely obese children. An even better option would be to study cohorts of children losing and gaining weight so that, always within the limitations of the observational framework, the effect of weight loss on the relationship between ISEC and serum lipids could be at least tentatively be modeled.

## Declaration of interest

The authors declare that there is no conflict of interest that could be perceived as prejudicing the impartiality of the research reported.

## Funding

Supported by Progetti di Ricerca Corrente, Istituto Auxologico Italiano.
